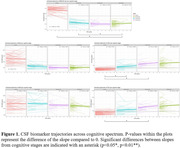# CSF biomarkers trajectories across the AD spectrum reveal the temporal dynamics of amyloid and tau

**DOI:** 10.1002/alz.091303

**Published:** 2025-01-09

**Authors:** Diederick Martijn de Leeuw, Calvin Trieu, Eleonora M. Vromen, Inge M.W. Verberk, Elena Raluca Blujdea, Charlotte Teunissen, Yolande A.L. Pijnenburg, Wiesje M. van der Flier, Argonde C. van Harten, Pieter Jelle Visser, Betty M. Tijms

**Affiliations:** ^1^ Alzheimer Center Amsterdam, Neurology, Vrije Universiteit Amsterdam, Amsterdam UMC location VUmc, Amsterdam Netherlands; ^2^ Amsterdam Neuroscience, Neurodegeneration, Amsterdam Netherlands; ^3^ Department of Clinical Chemistry, Neurochemistry Lab and Biobank, Amsterdam Neuroscience, Amsterdam UMC, Location VUmc, Amsterdam Netherlands; ^4^ Alzheimer Center Amsterdam, Neurology, Vrije Universiteit Amsterdam, Amsterdam UMC, Amsterdam Netherlands; ^5^ Amsterdam Neuroscience, Neurodegeneration, Amsterdam, Noord‐Holland Netherlands; ^6^ Alzheimer Centrum Limburg, Maastricht University, Maastricht Netherlands; ^7^ Department of Neurobiology, Care Sciences and Society, Division of Neurogeriatrics, Karolinska Institutet, Stockholm Sweden

## Abstract

**Background:**

The Alzheimer’s disease (AD) research framework proposes a biological definition of the disease. Still, little is known about longitudinal dynamics of core AD biomarker trajectories and how they map to each stage on the clinical spectrum.

**Methods:**

Participants with abnormal amyloid across the cognitive spectrum (i.e. preclinical AD, prodromal AD and AD dementia) and cognitively unimpaired controls with initially normal CSF markers were included from Amsterdam Alzheimer Center studies when they had at least one repeated CSF sample available. In all 452 samples, we measured Aß1‐42, Aß 1‐40, Aß1‐42/Aß1‐40 ratio, total tau (tTau), phosphorylated tau (pTau) with the Lumipulse multiplex. We tested longitudinal changes in all markers with linear mixed models including random intercepts and slopes, adjusting for age and sex. Each model included an interaction term of time and clinical stage. The Emmeans package in R was used to extract stage specific estimates.

**Results:**

By definition, all controls had higher (i.e., normal) amyloid markers and lower tau markers compared to subjects with preclinical, prodromal and AD dementia at baseline (p<0.001; Figure 1). Prodromal AD and dementia had the highest levels of tTau and pTau (all p<0.001) and the lowest levels of Aß1‐42/1‐40 ratio (all p<0.001), but were similar between both stages. Over 4±1sd years, Aß1‐42/1‐40 ratios decreased in controls and preclinical AD (p<0.001 and p=0.01 respectively), but remained unchanged in prodromal AD and dementia. CSF tTau and pTau increased over time in controls, preclinical and prodromal AD (all p<0.05), but did not in the dementia stage. Increases in pTau levels over time were steeper in both preclinical and prodromal AD than in controls (p<0.05) with 10% of controls reaching abnormal levels. Increases in tTau levels were steeper in prodromal AD only compared to controls (p<0.05).

**Conclusion:**

In this sample, we found that amyloid shifts towards abnormal levels in controls, becomes more abnormal in preclinical AD, and stabilizes in MCI and dementia. Tau markers become more abnormal in preclinical and prodromal AD only, suggesting that increase in CSF tau requires abnormal amyloid. This suggests that interventions targeting amyloid optimally happen before CSF amyloid becomes abnormal to prevent tau accumulation.